# Discovery and Genome Annotation of *Arthrobacter* Bacteriophages LastNadiia and Mireles Isolated from Soil in Eastern Texas

**DOI:** 10.17912/micropub.biology.002387

**Published:** 2026-10-29

**Authors:** Kateryna Voroshylo, Neyda Figueroa, Karina Santana, Traniece Bizzell, Gerardo Cerda, Lesly Calixto, Makayla Howell, Payten Belt, Shabnam Fathima, Venugopalan Cheriyath, Archana Srinivas, Hyun-Joo Nam

**Affiliations:** 1 Department of Biological and Environmental Sciences, East Texas A&M, Commerce, TX, United States

## Abstract

LastNadiia and Mireles are novel bacteriophages of *Arthrobacter globiformis* isolated from eastern Texas soil. Both exhibit siphoviral morphology and belong to the recently established cluster FR. Their genomes display the characteristic cluster FR organization, with predominantly forward-transcribed genes in the first half and reverse-transcribed genes in the second half. Neither genome contains identifiable lysogeny-associated genes, suggesting a lytic lifestyle.

**Figure 1. Transmission electron micrographs of LastNadiia and Mireles:** Transmission electron micrographs of LastNadiia (A) and Mireles (B), negatively stained with 1% uranyl acetate, showing siphovirus morphology. Scale bars represent 200 nm, with tick marks indicating 20 nm intervals. Table: Genome and Sequencing Characteristics of Bacteriophages LastNadiia and Mireles.

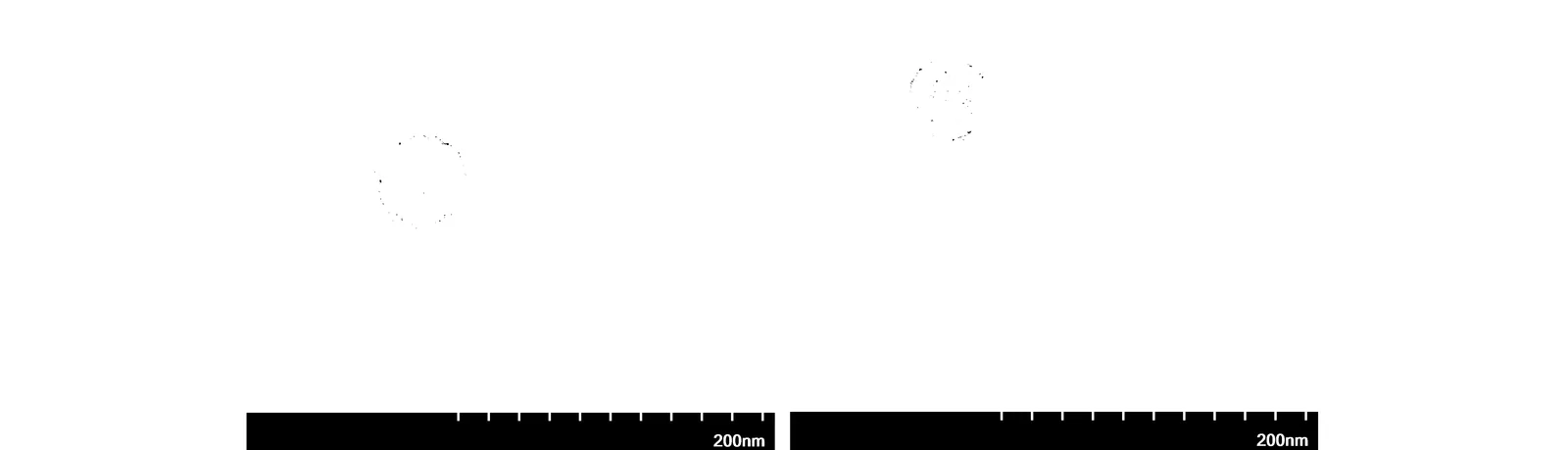



**Table:** 

Phage name	LastNadiia	Mireles
Genome length (bp)	41591	41147
Sequencing coverage (fold)	1473	1115
Number of sequencing reads	825,389	674,048
Genome end characteristics	3' sticky overhang (10 bases)	3' sticky overhang (10 bases)
Number of predicted protein coding genes	66	68
GC content (%)	58.5	58.6
Cluster	FR	FR
GenBank Accession Number	PZ492792	PZ492800
SRA Accession Number	SRX32748221	SRX32748206

## Description

Continued efforts to isolate and characterize novel bacteriophages have expanded our understanding of phage diversity, evolution, and genome organization (Heller et al., 2024). Here, we report the isolation, morphological characterization, and genome sequencing and annotation of LastNadiia and Mireles, two novel bacteriophages with predicted lytic lifestyles that infect *Arthrobacter globiformis* B-2979. LastNadiia and Mireles were isolated from soil samples obtained in eastern Texas. The sample containing phage LastNadiia was collected on the East Texas A&M University campus near Charles S. Garvin Lake (33.24377° N, 95.91124° W), whereas phage Mireles was isolated from a sample collected from a natural stormwater basin in Caddo Mills, Texas, USA (33.04428° N, 96.2313° W). The phages were isolated following the established protocol (Zorawik et al., 2024). Briefly, soil samples were suspended in PYCa medium, vortexed, and filtered through a 0.22-µm membrane. The filtrates were inoculated with *Arthrobacter globiformis* B-2979 and incubated at 30°C for 48 h. Following enrichment, the cultures were filtered, and the filtrates were plated with *A. globiformis* B-2979 using the soft-agar overlay method. Initial plaques were small and heterogeneous; after three rounds of plaque purification, both LastNadiia and Mireles formed clear, well-defined plaques ranging from 1.5 to 3 mm in diameter (n = 6). Transmission electron microscopy (TEM) of high-titer lysates was performed using a Hitachi HT7800 microscope operated at an accelerating voltage of 120 kV and equipped with an AMT Nanosprint15 B camera, bright-field STEM, and an Oxford Xplore EDS detector. TEM images showed that both phages exhibit siphovirus morphology, characterized by an icosahedral capsid and a long, flexible, noncontractile tail ( Fig. 1 A and B). TEM analyses showed that LastNadiia has a capsid diameter of approximately 60 nm and a tail length of 122 nm (n = 1), while Mireles has a 53 nm head diameter and a 120 nm tail length (n = 1). Genomic DNA was extracted from high-titer lysates using the Promega Wizard DNA Clean-Up Kit. Sequencing libraries were prepared with the NEBNext Ultra II DNA Library Prep Kit and sequenced on an Illumina NextSeq 1000 (XLEAP-P1 kit) at the Pittsburgh Bacteriophage Institute. Raw reads were trimmed with cutadapt 4.7 (using the option: –nextseq-trim 30) and filtered with skewer 0.2.2 (using the options: -q 20 -Q 30 -n -l 50) prior to assembly (Jiang et al., 2014; Martin, 2011; Wick et al., 2017). Illumina shotgun sequencing reads for both LastNadiia and Mireles were assembled using Newbler v2.9 (Miller et al., 2010), producing a single contig for each genome. Assembly completeness, accuracy, and genomic termini were verified using Consed v29 (Chung et al., 2017; Gordon et al., 1998; Russell, 2018). The LastNadiia and Mireles genomes are 41,591 bp and 41,147 bp, with GC contents of 58.5% and 58.6%, respectively. Both genomes contain identical 10-base 3′ cohesive ends (5′-TTTACGGCAT-3′). A summary of the sequencing results and genomic characteristics of LastNadiia and Mireles is provided in Table 1. Genome annotation of LastNadiia and Mireles began with automated gene prediction using Glimmer v3.02 (Delcher et al., 2007) and GeneMark v2.5 (Besemer and Borodovsky, 2005), followed by manual refinement in PECAAN v2.0 (http://discover.kbringsgd.org) which integrates multiple annotation tools. Gene start sites were evaluated using Phamerator (Actino_draft database v578) (Cresawn et al., 2011) and Starterator v627 (phages.wustl.edu/starterator/). Putative gene functions were assigned based on BLASTp v2.2.26 searches against the Actinobacteriophage and NCBI databases (Boratyn et al., 2013), and HHPred searches against the PDB_mmCIF70, pfam-A-v37.4, NCBI_Conserved_Domains (CD), and SCOPe70_2.08 and tRNAscan-SE databases (Soding et al., 2005). Membrane proteins were predicted with DeepTMHMM v1.0.42 (Hallgren et al., 2022). No tRNA genes were identified in either genome using Aragorn v1.2.38 (Chan and Lowe, 2019; Laslett and Canback, 2004). All software was run with default settings. The LastNadiia genome contains 66 predicted protein-coding genes, 35 of which were assigned putative functions. Of the remaining hypothetical genes, one was classified as an orpham. In Mireles, 68 protein-coding genes were predicted, including 33 with putative functional assignments. Among the remaining hypothetical genes, five were identified as orphams. No immunity-related genes were detected in either genome, consistent with the predicted lytic lifestyle of both phages. LastNadiia and Mireles were assigned to the cluster FR based on gene content similarity of at least 35% to phages in the Actinobacteriophage database, phagesdb (Pope et al., 2017; Russell and Hatfull, 2017). The cluster FR is a recently established cluster, with its first members reported in 2024, and currently comprises 11 fully annotated bacteriophages (Robinson et al., 2025). All FR phages exhibit a conserved genome organization, with most forward-transcribed genes located in the first half of the genome and predominantly reverse-transcribed genes in the second half. A single forward-transcribed gene is embedded within the reverse-transcribed region, followed by a terminal forward-transcribed gene at the end of the genome. These findings expand our understanding of *Arthrobacter* phages in the recently established cluster FR by defining key genomic and morphological features of LastNadiia and Mireles.
